# Thyroid hormone signaling specifies cone subtypes in human retinal organoids

**DOI:** 10.1126/science.aau6348

**Published:** 2018-10-12

**Authors:** Kiara C. Eldred, Sarah E. Hadyniak, Katarzyna A. Hussey, Boris Brenerman, Ping-Wu Zhang, Xitiz Chamling, Valentin M. Sluch, Derek S. Welsbie, Samer Hattar, James Taylor, Karl Wahlin, Donald J. Zack, Robert J. Johnston

**Affiliations:** 1 Department of Biology, Johns Hopkins University, 3400 N. Charles Street, Baltimore, MD 21218, USA.; 2 Wilmer Eye Institute, Johns Hopkins University School of Medicine, Baltimore, MD 21287, USA.; 3 Shiley Eye Institute, University of California, San Diego, La Jolla, CA 92093, USA.; 4 National Institute of Mental Health, National Institutes of Health, Bethesda, MD 20892, USA.; 5 Department of Computer Science, Johns Hopkins University, 3400 N. Charles Street, Baltimore, MD 21218, USA.; 6 Department of Molecular Biology and Genetics, Johns Hopkins University School of Medicine, Baltimore, MD 21287, USA.; 7 Department of Neuroscience, Johns Hopkins University School of Medicine, Baltimore, MD 21287, USA.; 8 Institute of Genetic Medicine, Johns Hopkins University School of Medicine, Baltimore, MD 21287, USA.

## Abstract

**INTRODUCTION:**

Cone photoreceptors in the human retina enable daytime, color, and high-acuity vision. The three subtypes of human cones are defined by the visual pigment that they express: blue-opsin (short wavelength; S), green-opsin (medium wavelength; M), or red-opsin (long wavelength; L). Mutations that affect opsin expression or function cause various forms of color blindness and retinal degeneration.

**RATIONALE:**

Our current understanding of the vertebrate eye has been derived primarily from the study of model organisms. We studied the human retina to understand the developmental mechanisms that generate the mosaic of mutually exclusive cone subtypes. Specification of human cones occurs in a two-step process. First, a decision occurs between S versus L/M cone fates. If the L/M fate is chosen, a subsequent choice is made between expression of L- or M-opsin. To determine the mechanism that controls the first decision between S and L/M cone fates, we studied human retinal organoids derived from stem cells.

**RESULTS:**

We found that human organoids and retinas have similar distributions, gene expression profiles, and morphologies of cone subtypes. During development, S cones are specified first, followed by L/M cones. This temporal switch from specification of S cones to generation of L/M cones is controlled by thyroid hormone (TH) signaling. In retinal organoids that lacked thyroid hormone receptor β (*Thrβ*), all cones developed into the S subtype. *Thrβ* binds with high affinity to triiodothyronine (T3), the more active form of TH, to regulate gene expression. We observed that addition of T3 early during development induced L/M fate in nearly all cones. Thus, TH signaling through *Thrβ* is necessary and sufficient to induce L/M cone fate and suppress S fate. TH exists largely in two states: thyroxine (T4), the most abundant circulating form of TH, and T3, which binds TH receptors with high affinity. We hypothesized that the retina itself could modulate TH levels to control subtype fates. We found that deiodinase 3 (*DIO3*), an enzyme that degrades both T3 and T4, was expressed early in organoid and retina development. Conversely, deiodinase 2 (*DIO2*), an enzyme that converts T4 to active T3, as well as TH carriers and transporters, were expressed later in development. Temporally dynamic expression of TH-degrading and -activating proteins supports a model in which the retina itself controls TH levels, ensuring low TH signaling early to specify S cones and high TH signaling later in development to produce L/M cones.

**CONCLUSION:**

Studies of model organisms and human epidemiology often generate hypotheses about human biology that cannot be studied in humans. Organoids provide a system to determine the mechanisms of human development, enabling direct testing of hypotheses in developing human tissue. Our studies identify temporal regulation of TH signaling as a mechanism that controls cone subtype specification in humans. Consistent with our findings, preterm human infants with low T3 and T4 have an increased incidence of color vision defects. Moreover, our identification of a mechanism that generates one cone subtype while suppressing the other, coupled with successful transplantation and incorporation of stem cell-derived photoreceptors in mice, suggests that the promise of therapies to treat human diseases such as color blindness, retinitis pigmentosa, and macular degeneration will be achieved in the near future. ■

## Summary

The mechanisms underlying specification of neuronal subtypes within the human nervous system are largely unknown. The blue (S), green (M), and red (L) cones of the retina enable high-acuity daytime and color vision. To determine the mechanism that controls S versus L/M fates, we studied the differentiation of human retinal organoids. Organoids and retinas have similar distributions, expression profiles, and morphologies of cone subtypes. S cones are specified first, followed by L/M cones, and thyroid hormone signaling controls this temporal switch. Dynamic expression of thyroid hormone–degrading and –activating proteins within the retina ensures low signaling early to specify S cones and high signaling late to produce L/M cones. This work establishes organoids as a model for determining mechanisms of human development with promising utility for therapeutics and vision repair.

## Graphical Abstract

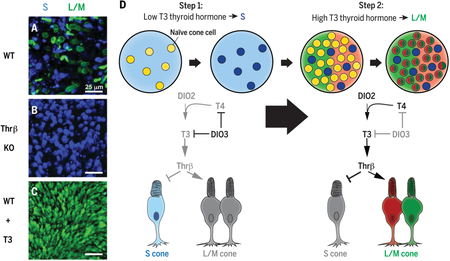

**Temporally regulated TH signaling specifies cone subtypes.** (**A**) Embryonic stem cell-derived human retinal organoids [wild type (WT)] generate S and L/M cones. Blue, S-opsin; green, L/M-opsin. (**B**) Organoids that lack thyroid hormone receptor β (*Thrβ* KO) generate all S cones. (**C**) Early activation of TH signaling (WT + T3) specifies nearly all L/M cones. (**D**) TH-degrading enzymes (such as DIO3) expressed early in development lower TH and promote S fate, whereas TH-activating regulators (such as DIO2) expressed later promote L/M fate.

Cone photoreceptors in the human retina enable daytime, color, and high-acuity vision (1). The three subtypes of human cones are defined by the visual pigment that they express: blue-opsin (short wavelength; S), green-opsin (medium wavelength; M), or red-opsin (long wavelength; L) (2). Specification of human cones occurs in a two-step process. First, a decision occurs between S versus L/M cone fates (Fig. 1A). If the L/M fate is chosen, a subsequent choice is made between expression of L- or M-opsins (3–6). Mutations that affect opsin expression or function cause various forms of color blindness and retinal degeneration (7–9). Great progress has been made in our understanding of the vertebrate eye through the study of model organisms. However, little is known about the developmental mechanisms that generate the mosaic of mutually exclusive cone subtypes in the human retina. We studied the specification of human cone subtypes using human retinal organoids differentiated from stem cells (Fig. 1, D to K).

Human retinal organoids generate photoreceptors that respond to light (10–14). We found that human organoids recapitulate the specification of cone subtypes observed in the human retina, including the temporal generation of S cones followed by L and M cones. Moreover, we found that this regulation is controlled by thyroid hormone signaling, which is necessary and sufficient to control cone subtype fates through the nuclear hormone receptor thyroid hormone receptor β (Thr*β*). Expression of thyroid hormone-regulating genes suggests that retina-intrinsic temporal control of thyroid hormone levels and activity governs cone subtype specification. Whereas retinal organoids have largely been studied for their promise of therapeutic applications (15), our work demonstrates that human organoids can also be used to reveal fundamental mechanisms of human development.

## Specification of cone cells in organoids recapitulates development in the human retina

**Fig. 1.**
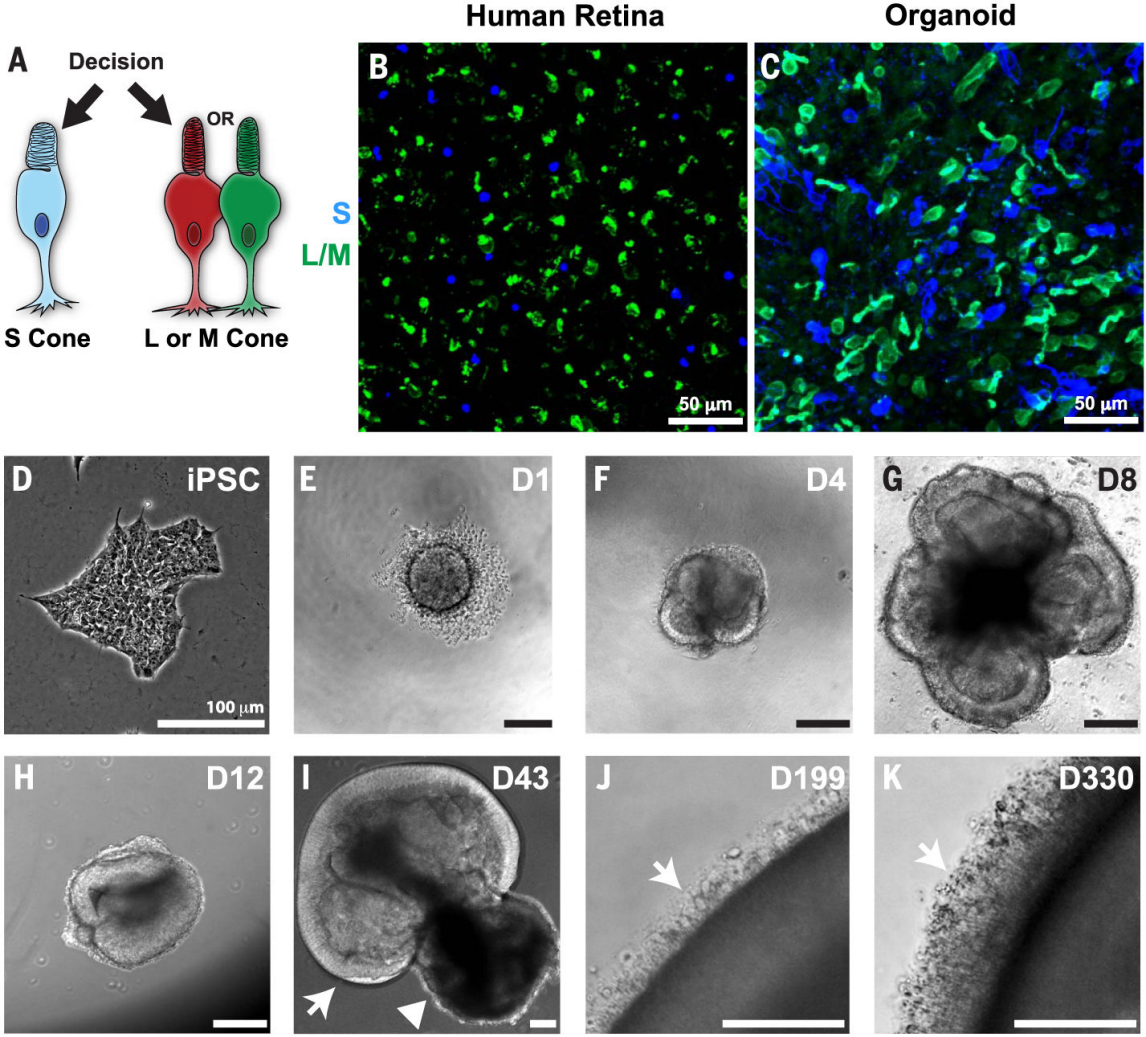
S and L/M cone generation in human retinal organoids. (**A**) Decision between S and L/M cone subtype fate. (**B** and **C**) S-opsin (blue) and L/M-opsin (green). (B) Human adult retina age 53. (C)iPSC-derived organoid, day 200 of differentiation. (**D** to **K**) Bright-field images of organoids derived from iPSCs. (D) Undifferentiated iPSCs. (E) Day 1, aggregation. (F) Day 4, formation of neuronal vesicles. (G) Day 8, differentiation of retinal vesicles. (H) Day 12, manual isolation of retinal organoid. (I) Day 43, arrow indicates developing retinal tissue, and arrowhead indicates developing retinal pigment epithelium. (J) Day 199, arrow indicates outer segments. (K) Day 330, arrow indicates outer segments.

**Fig. 2.**
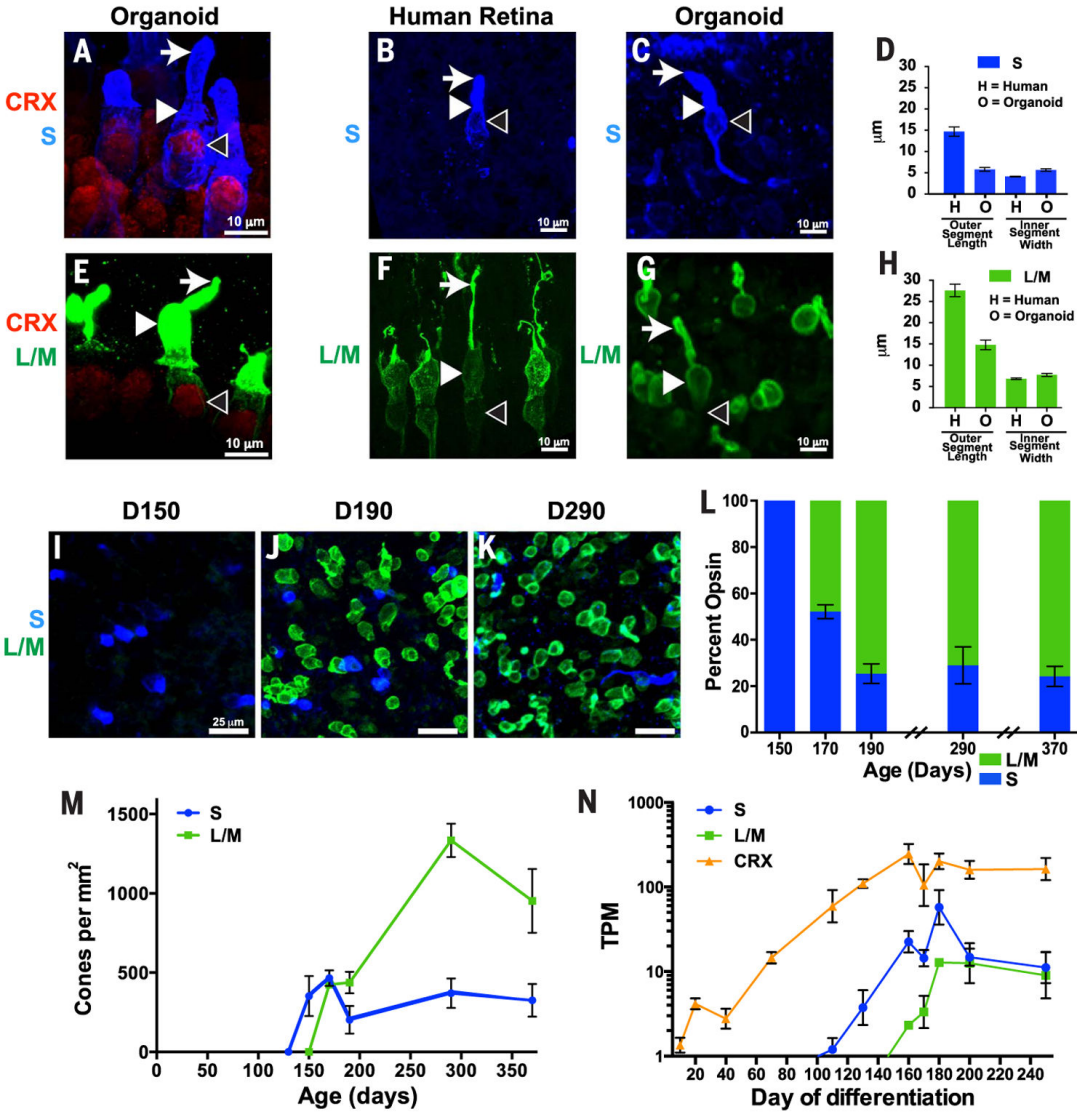
Human cone subtype specification is recapitulated in organoids. (**A** to **K**) S-opsin (blue) and L/M-opsin (green) were examined in human iPSC-derived organoids [(A), (C) to (E), and (G) to (M)] and human retinas [(B), (D), (F), and (H)]. [(A) to (C) and (E) to (G)] Arrows indicate outer segments, solid arrowheads indicate inner segments, and open arrowheads indicate nuclei. [(A) and (E)] CRX (a general marker of photoreceptors) is expressed in S cones and L/M cones. [(B) to (D)] S cones display short outer segments and thin inner segments in both human retinas and organoids. [(F) to (H)] L/M cones display long outer segments and wide inner segments in both human retinas and organoids. [(D) and (H)] Quantification of outer segment lengths and inner segment widths (adult retina, L/M, *n* = 13 cones, S, *n* = 10 cones; organoid, L/M, *n* = 35 cones, S, *n* = 42 cones). [(I) to (N)] S cones are generated before L/M cones in organoids. (**L**) Ratio of S:L/M cones during organoid development. (**M**) Density of S and L/M cones during organoid development. (**N**) *S-opsin* expression precedes *L/M-opsin* expression in human iPSC-derived organoids. *CRX* expression starts before opsin expression. TPM, transcripts per kilobase million.

We compared features of cone subtypes in human organoids with those of adult retinal tissue. Adult human retinas and organoids at day 200 of differentiation displayed similar ratios of S to L/M cones as indicated by expression of S- or L/M-opsins (adult, S = 13%, L/M = 87%; organoid, S = 29%, L/M = 71%) (Fig. 1, B and C, and fig. S1A). The difference in the ratios is likely due to the immaturity of the organoid at ~6 months compared with the terminally differentiated adult retina. We examined L/M cones with an antibody that recognizes both L- and M-opsin proteins because of their extremely high similarity. Both S and L/M cones expressed the cone-rod-homeobox transcription factor (CRX), a critical transcription factor for photoreceptor differentiation (Fig. 2, A and E) (16–18), indicating proper fate specification in organoids. Additionally, cones in organoids and retinas displayed similar morphologies, with L/M cones that had longer outer segments and wider inner segments than those of S cones (Fig. 2, B to D and F to H) (19). The outer segments of cones were shorter in organoids than in adult retinas, which is consistent with postnatal maturation (Fig. 2, D and H) (20). Thus, cone subtypes in human retinal organoids displayed distributions, gene expression patterns, and morphologies similar to those of cones of the human retina.

We next examined the developmental dynamics of cone subtype specification in organoids. In the human retina, S cones are generated during fetal weeks 11 to 34 (days 77 to 238), whereas L/M cones are specified later, during fetal weeks 14 to 37 (days 98 to 259) (21, 22). We tracked the ratios and densities of S and L/M cones in organoids by means of antibody staining over 360 days of differentiation. Cones expressing S-opsin were first observed at day 150 (Fig. 2, I, L, and M). The density of S cones leveled off at day 170 (Fig. 2M), at the time point when cones expressing L/M-opsin began to be observed (Fig. 2, J to M). The population of L/M cones increased dramatically until day 300 (Fig. 2, K to M), when they reached a steady-state density. The 20-day difference between S- and L/M-opsin expression onset in retinal organoids is similar to the 20-day difference observed in the appearance of S and L/M cones in the fetal retina (21). These observations show a temporal switch from S cone specification to L/M cone specification during retinal development.

We next conducted RNA sequencing (RNA-seq) through 250 days of induced pluripotent stem cell (iPSC)-derived organoid development. We found that *S-opsin* RNA was expressed first at day 111 and leveled off at day 160, whereas *L/M-opsin* RNA was expressed at day 160 and remained steady after day 180, which is consistent with the timeline of photoreceptor maturation in organoids and fetal retinas (Fig. 2N and fig. S1B). Moreover, *CRX* RNA and CRX protein were expressed before opsins in organoids, which is similar to human development (Fig. 2N and fig. S1, B to G) (23). Thus, human organoids recapitulate many aspects of the developmental timeline of cone subtype specification observed in human retinas, providing a model system with which to uncover the mechanisms of these developmental changes.

## Thyroid hormone signaling and the temporal switch between S and L/M fate specification

**Fig. 3.**
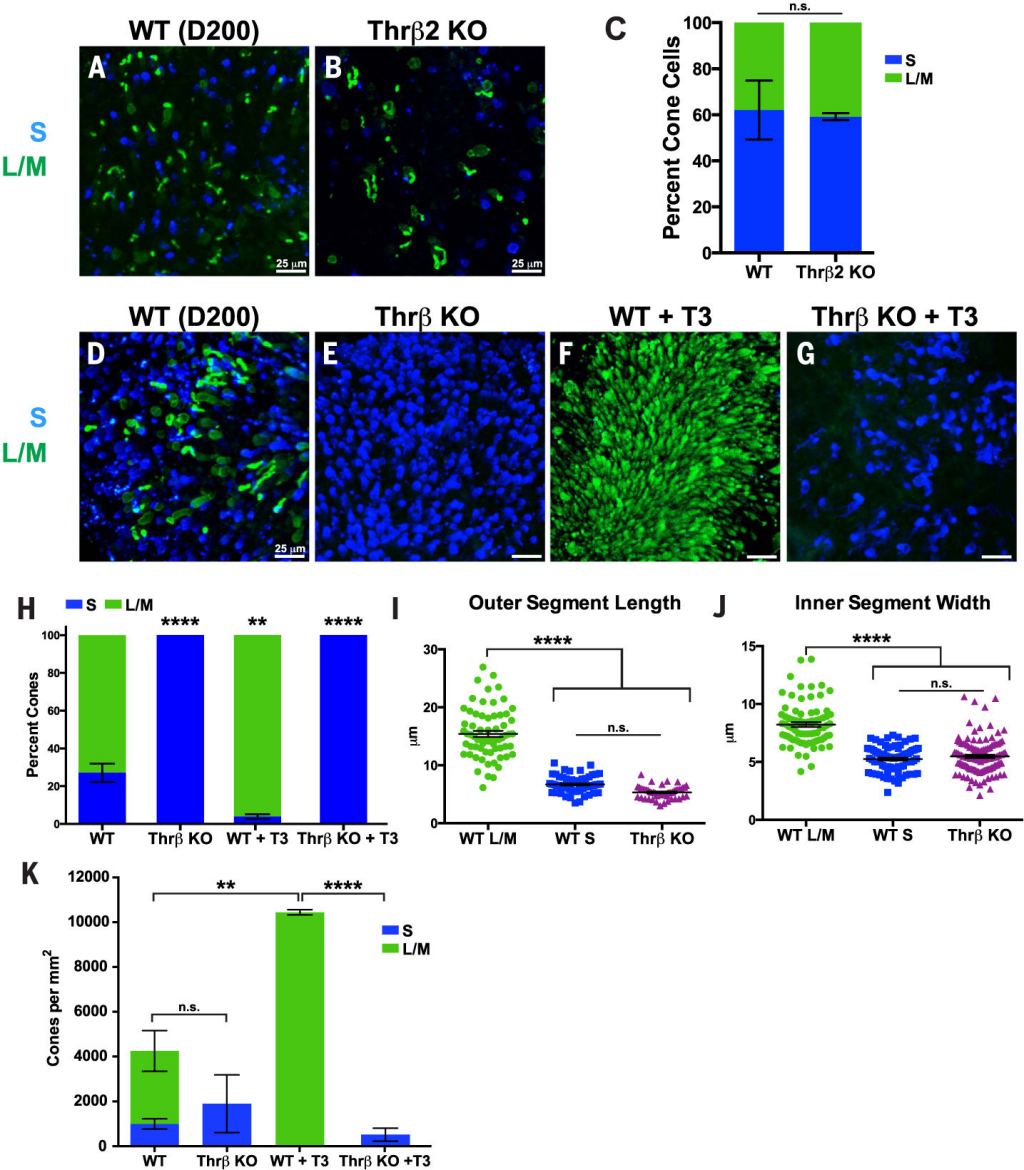
Thyroid hormone signaling is necessary and sufficient for the temporal switch between S and L/M fate specification. (**A** to **K**) S-opsin (blue) and L/M-opsin (green) were examined in human ESC-derived organoids. (A) Wild-type (WT). (B) *Thrβ2* early termination mutant (*Thrβ2* KO). (C) Quantification of (A) and (B) (WT, *n* = 3 organoids; *Thrβ2* KO, *n* = 3 organoids). (D) WT. (E) *Thrβ* KO. (F) WT treated with 20 nM T3 (WT + T3). (G) *Thrβ* KO treated with 20 nM T3 (*Thrβ* KO + T3). (H) Quantification of (D) to (G) (WT, *n* = 9 organoids; *Thrβ* KO, *n* = 3 organoids; WT + T3, *n* = 6 organoids; *Thrβ* KO + T3, *n* = 3 organoids. Tukey’s multiple comparisons test: WT versus *Thrβ* KO, *P* < 0.0001; WT versus WT + T3, *P* < 0.01; WT + T3 versus *Thrβ* KO + T3, *P* < 0.0001). (I) Length of outer segments. WT, L/M *n* = 66 cells; WT, S *n* = 66 cells; *Thrβ* KO, *n* = 50 cells (Tukey’s multiple comparisons test, WT L/M versus WT S, *P* < 0.0001; WT L/M versus *Thrβ* KO, *P* < 0.0001; WT S versus *Thrβ* KO, not significantly different). (J) Width of inner segments. WT, L/M *n* = 78 cells; WT, S *n* = 78 cells; *Thrβ* KO, *n* = 118 cells (Tukey’s multiple comparisons test, WT L/M versus WT S, *P* < 0.0001; WT L/M versus *Thrβ* KO, *P* < 0.0001; WT S versus *Thrβ* KO, not significantly different). (K) T3 acts through *Thrβ* to increase total cone number. Quantification of density of S and L/M cones; WT, *n* = 6 organoids; *Thrβ* KO, *n* = 3 organoids; WT + T3, *n* = 3 organoids; *Thrβ* KO + T3, *n* = 3 organoids (Tukey’s multiple comparisons test between total cone numbers, WT versus *Thrβ* KO, not significantly different; WT versus WT + T3, *P* < 0.01; WT + T3 versus *Thrβ* KO + T3, *P* < 0.0001).

Seminal work in mice identified Thr*β*2 as a critical regulator of cone subtype specification: *Thrβ2* mutants display a complete loss of M-opsin expression and a complete gain of S-opsin expression in cone photoreceptors (*24–26*). Similar roles for *Thrβ2* have been characterized in other organisms with highly divergent cone patterning (27–29). Additionally, rare human mutations in *Thrβ2* are reported to alter color perception, which is indicative of a change in the S-to-L/M cone ratio (30). To directly test the role of *Thrβ2* in human cone subtype specification, we used CRISPR/Cas9 in human embryonic stem cells (ESCs) to generate a homozygous mutation that resulted in early translational termination in the first exon of *Thrβ2* (fig. S2A). Surprisingly, organoids derived from these mutant stem cells displayed no differences in cone subtype ratio from genotypically wild-type organoids [wild type, S = 62%, L/M = 38%; *Thrβ2* knockout (KO), S = 59%, L/M = 41%; *P* = 0.83]. The S-to-L/M ratio is high for both wild-type controls and *Thrβ2* KO organoids, likely owing to variability in organoid differentiation. Thus, unlike previous suggestions based on other species, *Thrβ2* is dispensable for cone subtype specification in humans (Fig. 3, A to C).

Because *Thrβ2* alone is not required for human cone subtype specification, we reexamined data from Weiss *et. al* (30) and found that mis-sense mutations in exons 9 and 10 affected both *Thrβ2* and another isoform of the human *Thrβ* gene, *Thrβ1* (fig. S2A). Thus, we asked whether *Thrβ1* and *Thrβ2* together are required for cone subtype specification in humans. To completely ablate Thrβ function (Thr*β*1 and Thr*β*2), we used CRISPR/Cas9 in human ESCs to delete a shared exon that codes for part of the DNA binding domain of *Thrβ* (fig. S2A). *Thrβ* null mutant retinal organoids displayed a complete conversion of all cones to the S subtype (wild type, S = 27%, L/M = 73%; *Thrβ* KO, S = 100%, L/M = 0%; *P* < 0.0001) (Fig. 3, D to E and H). In these mutants, all cones expressed S-opsin and had the S cone morphology (Fig. 3, I and J). Thus, *Thrβ* is required to activate L/M and to repress S cone fates in the human retina.

Thrβ binds with high affinity to triiodothyronine (T3), the more active form of thyroid hormone, to regulate gene expression (31). Depletion or addition of T3 alters the ratios of S to M cones in rodents (25, 32, 33). Because L/M cones differentiate after S cones, we hypothesized that T3 acts through Thrβ late in retinal development to induce L/M cone fate and repress S cone fate. One prediction of this hypothesis is that addition of T3 early in development will induce L/M fate and repress S fate. To test this model, we added 20 nM T3 to ESC- and iPSC-derived organoids starting from days 20 to 50 and continued until day 200 of differentiation. We observed a dramatic conversion of cone cells to L/M fate (wild type, S = 27%, L/M = 73%; wild type + T3, S = 4%, L/M = 96%; *P* < 0.01) (Fig. 3, F and H, and fig. S2B). Thus, early addition of T3 is sufficient to induce L/M fate and suppress S fate.

To test whether T3 acts specifically through Thr*β* to control cone subtype specification, we differentiated *Thrβ* mutant organoids with early T3 addition. *Thrβ* mutation completely suppressed the effects of T3, generating organoids with only S cones (wild type + T3, S = 4%, L/M = 96%; *Thrβ* KO + T3, S = 100%, L/M = 0%; *P* < 0.0001) (Fig. 3, F to H). We conclude that T3 acts though Thr*β* to promote L/M cone fate and suppress S cone fate.

We confirmed the regulation of L/M-opsin expression through thyroid hormone signaling in a retinoblastoma cell line, which expresses L/M-opsin when treated with T3 (fig. S2, C and D) (34). T3-induced activation of *L/M-opsin* expression was suppressed upon RNA interference knockdown of *Thrβ* (fig. S2, E and F), which is similar to the suppression observed in human organoids.

In organoids, early T3 addition not only converted cone cells to L/M fate but also dramatically increased cone density (Fig. 3, F and K). Moreover, T3 acts specifically through Thr*β* to control cone density (Fig. 3, G and K). Early T3 addition may increase cone density by advancing and extending the temporal window of L/M cone generation.

Together, these results demonstrate that T3 signals though Thrβ to promote L/M cone fate and repress S cone fate in developing human retinal tissue.

## Dynamic expression of thyroid hormone–regulating genes during development

Our data suggest that temporal control of thyroid hormone signaling determines the S-versus-L/M cone fate decision, in which low signaling early induces S fate and high signaling late induces L/M fate. Thyroid hormone exists largely in two states: thyroxine (T4), the most abundant circulating form of thyroid hormone, and T3, which binds thyroid hormone receptors with high affinity (31, 35). Because the culture medium contains low amounts of T3 and T4, we hypothesized that the retina itself could modulate and/or generate thyroid hormone to control subtype fates.

Conversion of T4 to T3 occurs locally in target tissues to induce gene expression responses (36, 37). Deiodinases—enzymes that modulate the levels of T3 and T4—are expressed in the retinas of mice, fish, and chickens (29, 38–42). Therefore, we predicted that T3- and T4-degrading enzymes would be expressed during early human eye development to reduce thyroid hormone signaling and specify S cones, whereas T3-producing enzymes, carriers, and transporters would be expressed later in human eye development to increase signaling and generate L/M cones.

**Fig. 4.**
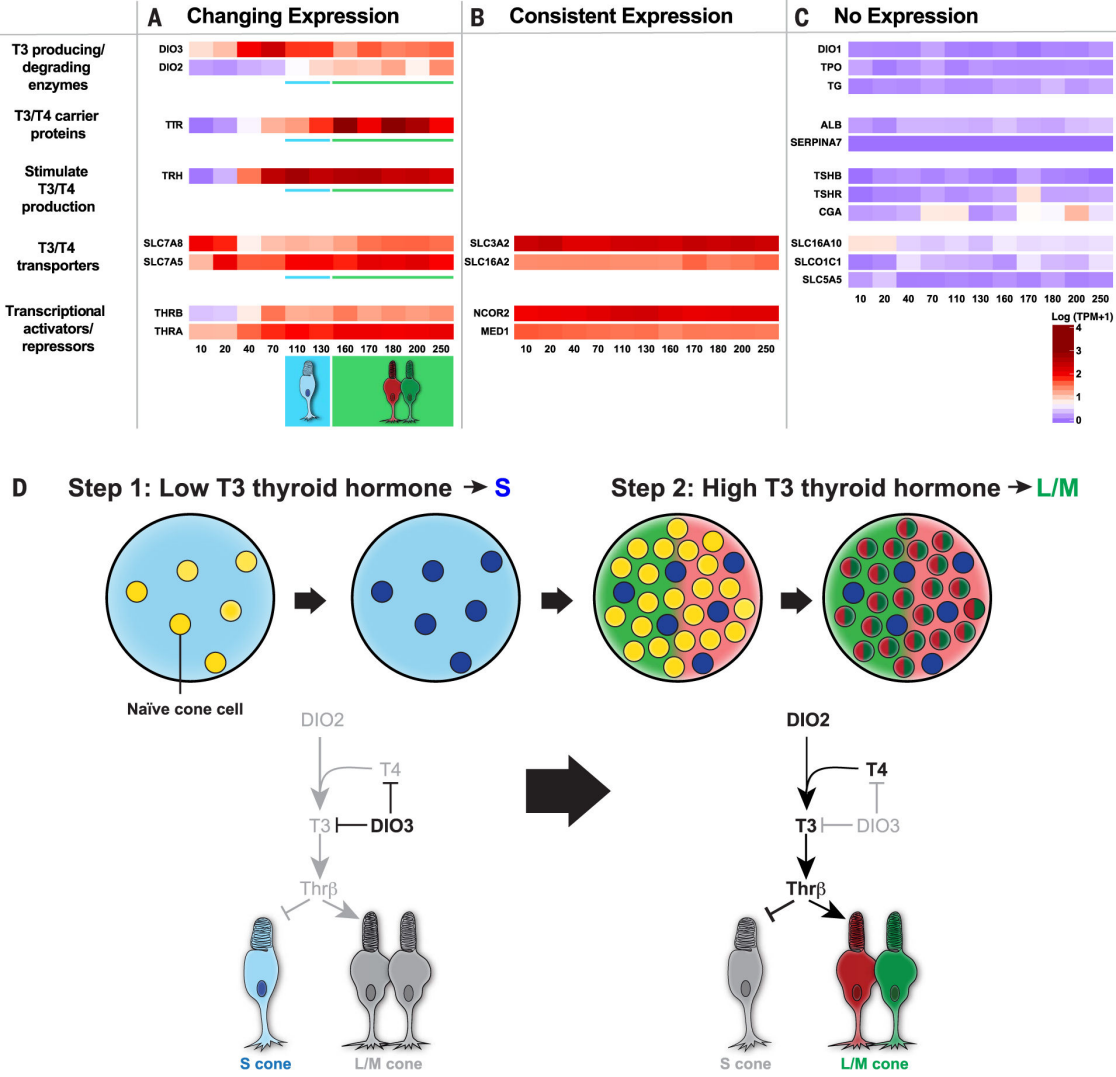
Dynamic expression of thyroid hormone signaling regulators during development. (**A** to **C**) Heat maps of log(TPM + 1) values for genes with (A) changing expression, (B) consistent expression, and (C) no expression. Numbers at the bottom of heat maps indicate organoid age in days. (**D**) Model of the temporal mechanism of cone subtype specification in humans. For simplicity, only the roles of DIO3 and DIO2 are illustrated. In step 1, expression of DIO3 degrades T3 and T4, leading to S cone specification. In step 2, expression of DIO2 converts T4 to T3 to signal Thrβ to repress S and induce L/M cone fate.

To test these predictions, we examined gene expression across 250 days of organoid development. The expression patterns of thyroid hormone–regulating genes were grouped into three classes: changing expression (Fig. 4A), consistent expression (Fig. 4B), or no expression (Fig. 4C). Deiodinase 3 (*DIO3*), an enzyme that degrades T3 and T4 (36), was expressed at high levels early in organoid development but at low levels later (Fig. 4A). Conversely, deiodinase 2 (*DIO2*), an enzyme that converts T4 to active T3 (36), was expressed at low levels early but then dramatically increased over time (Fig. 4A). We examined RNA-seq data from Hoshino *et. al* (23) and found that developing human retinas display similar temporal changes in expression of *DIO3* and *DIO2* (fig. S3A). Deiodinase 1 (*DIO1*), which regulates T3 and T4 predominantly in the liver and kidney (43), was not expressed in organoids or retinas (Fig. 4C and fig. S3C). Thus, the dynamic expression of *Dio3* and *Dio2* supports low thyroid hormone signaling early in development to generate S cones and high thyroid hormone signaling late to produce L/M cones.

Consistent with a role for high thyroid hormone signaling in the generation of L/M cones later in development, expression of transthyretin (*TTR*), a thyroid hormone carrier protein, increased during organoid and retinal development (Fig. 4A and fig. S3A) (23). By contrast, albumin (*ALB*) and thyroxine-binding globulin (*SERPINA7*), other carrier proteins of T3 and T4, were not expressed in organoids or retinas (Fig. 4C and fig. S3C) (23).

T3 and T4 are transported into cells via membrane transport proteins (44). The T3/T4 transporters *SLC7A5* and *SLC7A8* increased in expression during organoid differentiation (Fig. 4A). Additionally, two T3/T4 transporters, *SLC3A2* and *SLC16A2,* were expressed at high and consistent levels throughout organoid development (Fig. 4B). Other T3/T4 transporters (*SLC16A10, SLCO1C1,* and *SLC5A5*) were not expressed in organoids (Fig. 4C), suggesting tissue-specific regulation of T3/T4 uptake. We observed similar expression patterns of T3/T4 transporters in human retinas (fig. S3, A to C) (23).

We next examined expression of transcriptional activators and repressors that mediate the response to thyroid hormone. Consistent with *Thrβ* expression in human cones (45), expression of *Thrβ* in organoids increased with time as cone cells were specified (Fig. 4A). Expression of thyroid hormone receptor α (*Thrα*) similarly increased with time (Fig. 4A). Thyroid hormone receptor cofactors, corepressor *NCoR2* and coactivator *MED1,* were expressed at steady levels during organoid differentiation (Fig. 4B). Similar temporal expression patterns were observed in human retinas (fig. S3, A and B) (23). Thus, our data suggest that expression of Thrβ and other transcriptional regulators enables gene regulatory responses to differential thyroid hormone levels.

A complex pathway controls production of thyroid hormone. Thyrotropin-releasing hormone (TRH) is produced by the hypothalamus and other neural tissue. TRH stimulates release of thyroid-stimulating hormone α (CGA) and thyroid-stimulating hormone β (TSHβ) from the pituitary gland. CGA and TSHβ bind the thyroid-stimulating hormone receptor (TSHR) in the thyroid gland. T3 and T4 production requires thyroglobulin (TG), the substrate for T3/T4 synthesis, and thyroid peroxidase (TPO), an enzyme that iodinates tyrosine residues in TG (46). *TRH* was expressed in organoids and retinas, but the other players were not (Fig. 4, A to C, and fig. S3, A to C) (23, 47, 48), suggesting that the retina itself does not generate thyroid hormone; rather, it modulates the relative levels of T3 and T4 and expresses TRH to signal for thyroid hormone production in other tissues.

Therefore, the temporal expression of thyroid hormone signaling regulators supports our model that the retina intrinsically controls T3 and T4 levels, ensuring low thyroid hormone signaling early to promote S fate and high thyroid hormone signaling late to specify L/M fate (Fig. 4D).

Organoids provide a powerful system with which to determine the mechanisms of human development. Model organism and epidemiological studies generate important hypotheses about human biology that are often experimentally intractable. This work shows that organoids enable direct testing of hypotheses in developing human tissue.

Our studies identify temporal regulation of thyroid hormone signaling as a mechanism that controls cone subtype specification in humans. Consistent with our findings, preterm human infants with low T3/T4 have an increased incidence of color vision defects (49–52). Moreover, our identification of a mechanism that generates one cone subtype while suppressing the other, coupled with successful transplantation and incorporation of stem cell-derived photoreceptors in mice (53–56), suggests that the promise of therapies to treat human diseases such as color blindness, retinitis pigmentosa, and macular degeneration will be achieved in the near future.

## Materials and methods summary

### Cell lines

H7 ESC (WA07, WiCell) and episomal-derived EP1.1 iPSC lines were used for differentiation. WERI-Rb1 retinoblastoma cells were obtained from ATCC. Cell maintenance and organoid differentiation protocols are described in the supplementary materials.

### CRISPR mutations

All mutations were generated in H7 ESCs. Cells were modified to express an inducible Cas9 element. Plasmids for guide RNA (gRNA) transfection were generated by using the pSpCas9(BB)-P2A-Puro plasmid modified from the pX459_V2.0 plasmid (62988, Addgene) by replacing T2A with a P2A sequence. Mutations were confirmed with polymerase chain reaction sequencing. Gene diagrams of deletions are displayed in fig. S2A. Detailed transfection procedures, gRNA sequences, and homology arm sequences are included in the supplementary materials.

### Immunohistochemistry

Primary antibodies were used at the following dilutions: goat anti-SW-opsin (1:200 for organoids, 1:500 for human retinas) (Santa Cruz Biotechnology), rabbit anti-LW/MW-opsins (1:200 for organoids, 1:500 for human retinas) (Millipore), mouse anti-CRX (1:500) (Abnova), and mouse anti-Rhodopsin (1:500) (GeneTex). All secondary antibodies were Alexa Fluor–conjugated (1:400) and made in donkey (Molecular Probes). Detailed methods for fixation, microscopy, and image processing of organoids, retinas, and WERI-Rb1 cells are included in the supplementary materials.

### Organoid age

#### Opsin expression time course

EP1 iPSC-derived organoids for time course experiments were binned into 10-day increments for analysis. Organoids were binned into day 130 [actual day 129 (*n* = 3 organoids)], day 150 [actual day 152 (*n* = 4 organoids)], day 170 [actual day 173 (*n* = 2 organoids)], day 200 [actual days 194 to 199 (*n* = 7 organoids)], day 290 [actual day 291 (*n* = 3 organoids)], and day 360 [actual day 361 (*n* = 3 organoids)]. Quantifications of outer-segment lengths and inner-segment widths were measured in day 361 organoids (*n* = 3 organoids).

#### Opsin expression in different conditions

iCas9 H7 ESC–derived organoids for *Thrb2* KOs and controls were analyzed at day 200. Organoids for *Thrb* KO, control, and wild-type + T3 were analyzed at two time points: two organoids were taken at day 199 for each group, and one was taken at day 277 for each group. T3-treated organoids were taken at time points between day 195 and day 200 for different differentiations. For each treatment group and genotype, organoids were compared with control organoids grown in parallel.

#### RNA-seq time course

EP1 iPSC-derived organoids were analyzed at time points ranging from day 10 to day 250 of differentiation. We took samples at day 10 (*n* = 3 organoids), day 20 (*n* = 2 organoids), day 35 (*n* = 3 organoids), day 69 (*n* = 3 organoids), day 111 (*n* = 3 organoids), day 128 (*n* = 3 organoids), day 158 (*n* = 2 organoids), day 173 (*n* = 3 organoids), day 181 (*n* = 3 organoids), day 200 (*n* = 3 organoids), and day 250 (*n* = 3 organoids). RNA from individual organoids was extracted by using the Zymo Direct-zol RNA Microprep Kit (Zymo Research) according to manufacturer’s instructions. Libraries were prepared by using the Illumina TruSeq stranded mRNA kit and sequenced on an Illumina NextSeq 500 with single 200-base pair reads.

### RNA-seq time course analysis

Expression levels were quantified by using Kallisto (version 0.34.1) with the following parameters: “−b 100 −1 200 −s 10 −t 20–single”. The Gencode release 28 comprehensive annotation was used as the reference transcriptome (57). Transcripts per million (TPM) values (table S1) were then used to generate graphs in Prism and heatmaps in R by using ggplot2. The distributions of transcripts were plotted so as to identify the best low TPM cutoff (fig. S5A). The threshold was determined to be 0.7 log(TPM + 1)—5 TPM—and this value was used as an inflection point for the heatmaps. Heatmaps for fig. S3, A to C, were made similarly, by using CPM values from Hoshino *et. al* (fig. S5B) (23).

### Measurements and quantification

Measurements of retinal area and cell morphology were done by using ImageJ software. Quantifications and statistics (except for RNA-seq data) were done in GraphPad Prism, with a significance cutoff of 0.01. Statistical tests are listed in figure legends. All error bars represent the SEM.

## Supplementary Material

Supplemental Material
