# Supplementary material for: Thyroid hormone signaling specifies cone subtypes in human retinal organoids
Source: Science. Author manuscript; Available in PMC 2019 May 12. (PMC6249681; doi:10.1126/science.aau6348)
Supplement: Supplemental Material [file NIHMS993285-supplement-1.pdf]

www.sciencemag.org/content/362/6411/eaau6348/suppl/DC1

Supplementary Material for

Thyroid hormone signaling specifies cone subtypes in human retinal organoids

Kiara C. Eldred, Sarah E. Hadyniak, Katarzyna A. Hussey, Boris Brenerman, Ping-Wu Zhang, Xitiz Chamling, Valentin M. Sluch, Derek S. Welsbie, Samer Hattar, James Taylor, Karl Wahlin, Donald J. Zack, Robert J. Johnston Jr.*

*Corresponding author. Email: robertjohnston@jhu.edu

Published 12 October 2018, *Science* **362**, eaau6348 (2017)
DOI: 10.1126/science.aau6348

This PDF file includes:

Materials and Methods
Figs. S1 to S5
References

Other Supplementary Material for this manuscript includes the following:
(available at www.sciencemag.org/content/362/6411/eaau6348/suppl/DC1)

Table S1 as a separate Excel file

Materials and methods

Cell Lines

H7 ESC (WA07, WiCell) and episomal-derived EP1.1 iPSC lines were used (*58*). Pluripotency of EP1.1 was evaluated previously with antibodies for NANOG, OCT4, SOX2, SSEA4 (*12*). Stem cells were maintained in mTeSR1 (Stem Cell Technologies) on 1% (vol/vol) Matrigel-GFR™ (354230, BD Biosciences) coated dishes and grown in a 37°C HERAcell 150i incubator at 10% CO2 and 5% O2 incubator (Thermo Fisher Scientific). Cells were passaged every 3-6 days according to confluence as in Wahlin *et. al* (*12*). Cells were passaged with Accutase (SCR005, Sigma) for 7–10 minutes and dissociated to single cells. Cells in Accutase were added 1:2 to mTeSR1 plus 5 µM Blebbistatin (Bleb; B0560, Sigma), pelleted at 80 g for 5 minutes, and suspended in mTeSR1 plus Bleb and plated at 5,000 cells per well in a 6 well plate. After 48 hours, cells were fed with mTeSR1 (without Bleb) every 24 hours until the next passage. To minimize cell stress, no antibiotics were used.

WERI-Rb1 retinoblastoma cells were obtained from ATCC and maintained in RPMI + supplement media. Cells were grown in a 37°C HERAcell 150i 5% CO2 incubator (Thermo Fisher Scientific) and passaged every 3-4 days at ~1 x 10^5 – 2 x 10^6 cells/mL in uncoated flasks.

Cells were routinely tested for mycoplasma using MycoAlert (LT07, Lonza).

Cell Culture Media

Stem Cell media: mTeSR1 (StemCell Technologies)

E6 supplement: 970 ug/mL Insulin (11376497001, Roche), 535 ug/mL holo-transferrin (T0665, Sigma), 3.20 mg/mL L-ascorbic acid (A8960, Sigma), 0.7 ug/mL sodium selenite (S5261, Sigma).

BE6.2 media for early retinal differentiation: 2.5% E6 supplement (above), 2% minus vitamin A (12587010, Gibco), 1% Glutamax (35050061, Gibco), 1% NEAA (11140050, Gibco), 1mM Pyruvate (11360070, Gibco), and 0.87 mg/mL NaCl in DMEM (11885084, Gibco).

LTR (Long-Term Retina) media: 25% F12 (11765062, Gibco) with 2% B27 (17504044, Gibco), 10% heat inactivated FBS (16140071, Gibco), 1mM Sodium Pyruvate,1% NEAA, 1% Glutamax and 1 mM taurine (T-8691, Sigma) in DMEM (11885084, Gibco)

RPMI + supplement media: 10% heat inactivated FBS, 2.5% penicillin (30-002-CI, Corning) in RPMI Medium 1640 (Gibco).

Thyroid hormone treatment: For organoids, 20 nM T3 (T6397, Sigma) in LTR. This concentration is based on T3 treatment levels for mouse retinal explant experiments (*25*). For WERI-Rb1 cells, 100 nM T3 (T6397, Sigma) in RPMI + supplement media, similar to previous experiments inducing L/M opsin expression in this cell line (*34*).

Organoid differentiation

Organoids were differentiated from H7 WA07 ESCs or EP1.1 iPSCs as described in (*12*) with minor variations (**Fig. S4**).

Pluripotent stem cells were well-maintained, and only cultures with minimal to no spontaneous differentiation were used for aggregation. To aggregate, cells were passaged in Accutase at 37°C for 13 min to ensure complete dissociation. Cells were seeded in 50 ul of mTeSR1 at 3,000 cells/well into 96-well ultra-low adhesion round bottom Lipidure coated plates (51011610, NOF). Cells were placed in hypoxic conditions (10% CO2 and 5% O2) for 24 hours to enhance survival. Cells naturally aggregated by gravity over 24 hours.

On day 1, cells were moved to normoxic conditions (5% CO2). On days 1-3, 50 uLs of BE6.2 media containing 3 μM Wnt inhibitor (IWR1e: 681669, EMD Millipore) and 1% (v/v) Matrigel were added to each well. On days 4-9, 100 uLs of media were removed from each well, and 100 uLs of media were added. On days 4-5, BE6.2 media containing 3 μM Wnt inhibitor and 1% Matrigel was added. On days 6-7, BE6.2 media containing 1% Matrigel was added. On days 8-9, BE6.2 media containing 1% Matrigel and 100 nM Smoothened agonist (SAG: 566660, EMD Millipore) was added.

On day 10, aggregates were transferred to 15 mL tubes, rinsed 3X in DMEM (11885084, Gibco), and resuspended in BE6.2 with 100 nM SAG in untreated 10 cm polystyrene petri dishes. From this point on, media was changed every other day. Aggregates were monitored and manually separated if stuck together or to the bottom of the plate.

On days 13-16, LTR media with 100 nM SAG was added.

Between days 11 and 16, retinal vesicles were manually dissected using sharpened tungsten needles. After dissection, cells were transferred into 15 mL tubes and washed 2X with 5 mLs of DMEM.

On days 16-20, cells were maintained in LTR and washed 2X with 5 mLs of DMEM, before being transferred to new plates to wash off dead cells.

To increase survival and differentiation, 1 uM all-trans retinoic acid (ATRA; R2625; Sigma) was added to LTR medium from days 20-130. 10 μM Gamma-secretase inhibitor (DAPT: 565770, EMD Millipore) was added to LTR from days 28-42.

Organoids were grown at low density (10-20 per 10 cm dish, 2-3 per well in 6 well plate) to reduce aggregation.

CRISPR mutations

Cell line: All mutations were generated in H7 ESCs. Cells were modified to express an inducible Cas9 element. First, the puro-Cas9 donor plasmid was modified. The *Puromycin* N-acetyl transferase gene (puromycin–resistance gene) was replaced with Blasticidin S deaminase gene (blasticidin-resistance gene) using Xba I-Xho I restriction enzyme sites in the plasmid puro-Cas9 donor (58409, Addgene). This plasmid is referred to as the blast-Cas9 donor plasmid.

The integration of the targeting vectors into a previously genetically modified H7 human *ESC* line (*59*) was performed as follows: 0.25 million $H7^{Brn3B::tdTomato}$ ES cells at 50% confluence were transduced using a DNA-In

Stem kit (MTI-Globalstem, USA) with three plasmids (1ug each): Blast-Cas9 donor, M2rtTA donor (AAVS1-neo-M2rtTA: 60843, Addgene), and pSpCas9 (BB) plasmid (px459 v2.0: 62988, Addgene). gRNA sequences are listed below in the gRNA primer table. Cells were treated with Blasticidin (5ug/ml) and Geneticin (200ug/ml) for 5 days. Individual clones with both Blasticidin and Neomycin resistance survived, and were picked using sterile pipette tips and transferred to 96-well plates for clone identification. Positive clones carrying the correct insertion in both alleles were confirmed by PCR. Genotyping primers are listed below. Doxycycline induction was confirmed by qPCR and the verified clone iCas9 H7$^{Brn3B::tdTomato}$-24 was used for further experiments.

**Primers for Cas9**	**Primer Name**	**Primer Sequence**
Left arm	Left_ARM_F2	GGCCCTGGCCATTGTCACTT
	Cas9_Blast_R1	AGCAATTCACGAATCCCAAC
Right arm	Cas9_RARM_R1	CACCTTGTACTCGTCGGTGA
	Right_ARM_R1	GGAACGGGGCTCAGTCTGT
**Primers for rtTA**		
Left arm	Left_ARM_F2	GGCCCTGGCCATTGTCACTT
	rtTA_Neo_R1	GGCCATTTTCCACCATGATA
Right arm	rtTA_RARM_F2	GCTGATTATGATCCTGCAAGC
	Right_ARM_R1	GGAACGGGGCTCAGTCTGT

Cloning gRNA plasmids: Plasmids for gRNA transfection were generated using pSpCas9(BB)-P2A-Puro plasmid modified from the pX459_V2.0 plasmid (62988, Addgene) by replacing T2A with a P2A sequence. gRNAs were cloned into the vector following the Zhang Lab protocol: https://media.addgene.org/cms/filer_public/e6/5a/e65a9ef8-c8ac-4f88-98da-3b7d7960394c/zhang-lab-general-cloning-protocol.pdf

**gRNA Primer Name**	**Primer Sequence**
ThrB2_St_gRNA1_F	caccgAAAATACGCGTAATAATCAG
ThrB2_St_gRNA1_R	aaacCCTGATTATTACGCGTATTTTc
ThrB2_exon5_gRNA_F	caccGATACAGCGGTAGTGATACCCGG
ThrB2_exon5_gRNA_R	aaacCCGGGTATCACTACCGCTGTATC
AAVS1_gRNA_F	caccGGGGCCACTAGGGACAGGAT
AAVS1_gRNA_R	aaacATCCTGTCCCTAGTGGCCCc

Transfection and mutation identification

iCas9 stem cells were passaged in Accutase at 37°C for 13 min to ensure complete dissociation. Cells were seeded at 4 x 10^4 in 24 well plates for 24 hours in mTeSR with 5 μM Bleb. After 24 hours, media was removed and mTeSR was

added. Cells were transfected with 2.5 ul DNA-In Stem (GST-2130, Life Technologies), 250 ng gRNA plasmid PX459v2 containing the gRNA and Cas9-p2a-puromicin-resistance genes in 50 ul of Opti-MEM (31985062, Gibco). Cells were incubated for 24 hours, then media was removed and mTeSR and 1 ug Doxycycline (D9891, MilliporeSigma) were added. After 24 hours, media was removed and mTeSR, 1 ug Doxycycline, and 0.3-1 ug of puromycin were added. After 24 hours, media was removed, and cells were washed 1X with mTeSR, and mTeSR was then added to the well. Surviving cells were passaged at single cell density, individual colonies were isolated, and mutations were confirmed by PCR sequencing. Gene diagrams of deletions are displayed in **Fig. S2A**.

**Primer Name**	**Primer Sequence**
ThrB2_St_295_F	GTGCTTGGAAATCTTGATGTTCAC
ThrB2_St_296_R	GGTGGTGTTTATTCATCTTCCCTT
ThrB2_St_293_F	ATGTTCACAGAGTCCTTCAATCAC
ThrB2_St_297_R	CTGAACCAGGGAAACAAAATGAAC
ThrB2_exon5_285_F	GAAAACAGCCTGTGGTAGAGTAAA
ThrB2_exon5_287_R	GGTGTGAGCTATTTCTAAGGCATT
ThrB2_exon5_284_F	CTGTCTCCTCCAACACTGTAGATA
ThrB2_exon5_289_R	GAAATCCTGGGCCTATGTTAACTC
ThrB2_exon5_286_F	TTGCAGAAGTAAAGAAACCAGACA

Immunohistochemistry

Retinal organoids: Retinal organoids were fixed in fresh 4% formaldehyde and 5% sucrose in PBS for 1 hour. Tissue was rinsed 3X in 5% sucrose in PBS, then incubated at 4°C in 6.75% sucrose in PBS for 30 min, 12.5% sucrose in PBS for 30 min, and 25% sucrose for 2 hours-overnight. Organoids were incubated for 2 hours in blocking solution (0.2-0.3% Trition X-100, 2-4% donkey serum in PBS). Organoids were incubated with primary antibodies in blocking solution for 16-36 hours at 4°C. Organoids were washed 3X for 30 min in PBS, and then incubated with secondary antibodies in blocking solution for 2 hours at room temperature. Organoids were incubated in 300 nM DAPI in blocking solution for 10 min and washed 3X for 15 min in PBS. At the end of staining, organoids were mounted for imaging in slow fade (S36940, Thermo Fisher Scientific).

Retinas: Human retinas were obtained from the National Disease Research Interchange (NDRI). Human retinal tissue was fixed by the NDRI in 10% formalin within 12 hours post-mortem and stored at 4°C until dissection. Retinas were dissected and whole-mounted, then rinsed 3X in PBS for 20 min,

and blocked for 48 hours at 4°C in 0.3% Triton X-100 and 4% donkey serum. Retinas were stained with the same protocol as detailed above for organoids.

WERI-Rb1 cells: WERI-Rb1 cells were adhered to 0.01% w/v Poly-L-lysine slides for 1-2 hours at 37°C and 5% CO2 and then washed 1X in PBS. WERI-Rb1 cells were fixed in fresh 4% formaldehyde for 20 min. Slides were washed with PBS 3X, and then incubated for 2 hours in blocking solution. Primary antibodies were added at 4°C overnight. Slides were washed 3X in PBS and incubated in secondary antibodies for 2 hours at room temperature in blocking solution.

Antibodies

Primary antibodies were used at the following dilutions: goat anti-SW-opsin (1:200 for organoids, 1:500 for human retinas) (Santa Cruz Biotechnology), rabbit anti-LW/MW-opsins (1:200 for organoids, 1:500 for human retinas) (Millipore), and mouse anti-CRX (1:500) (Abnova), and mouse anti-Rhodopsin (1:500) (GeneTex). All secondary antibodies were Alexa Fluor-conjugated (1:400) and made in donkey (Molecular Probes).

Microscopy and image processing

Bright field images were acquired with a Nikon TE2000 or EVOS XL Core microscope. Fluorescent images were acquired with a Zeiss LSM710, LSM780, or LSM800 laser scanning confocal microscope. Confocal microscopy was performed with similar settings for laser power, photomultiplier gain and offset, and pinhole diameter. Maximum intensity projections of z-stacks (5–80 optical sections, 1.10 μm step size) were rendered to display all cones captured in a single organoid.

Organoid age

Opsin expression time course: EP1 iPSCs-derived organoids for time course experiments were binned into 10 day increments for analysis. Organoids were binned into day 130 (actual day 129 (n=3)), day 150 (actual day 152 (n=4)), day 170 (actual day 173 (n=2)), day 200 (actual days 194-199 (n=7)), day 290 (actual day 291 (n=3)), and day 360 (actual day 361 (n=3). Quantification of outer segment lengths and inner segment widths were measured in day 361 organoids (n=3).

Opsin expression in different conditions: iCas9 H7 ESC-derived organoids for Thrβ2 KOs and controls were analyzed at day 200. Organoids for Thrβ KO, control, and wild-type + T3 were analyzed at two time points: 2 organoids were taken at day 199 for each group, and one was taken at day 277 for each group. T3-treated organoids were taken at time points between day 195 and day 200 for different differentiations. For each treatment group and genotype, organoids were compared to control organoids grown in parallel.

RNA-Seq time course: EP1 iPSC-derived organoids were analyzed at time points ranging from day 10 to day 250 of differentiation. We took samples at day 10 (n=3), day 20 (n=2), day 35 (n=3), day 69 (n=3), day 111 (n=3), day 128 (n=3), day 158 (n=2), day 173 (n=3), day 181 (n=3), day 200 (n=3), and day 250

(n=3). RNA from individual organoids was extracted using the Zymo Direct-zol RNA Microprep Kit (Zymo Research) according to manufacturer's instructions. Libraries were prepared using the Illumina TruSeq stranded mRNA kit and sequenced on an Illumina NextSeq 500 with single 200 bp reads.

WERI-Rb1 siRNA and qPCR

Varying concentrations of WERI-Rb1cells were seeded onto 24-well plates with 500 uL of RPMI+Supplement. After ~24 hours, WERI-Rb1 cells were washed once with sterile DPBS (Gibco) and suspended in media with or without 100 nM T3. Negative control siRNA 1 (Thermo Fisher Scientific) or THRB ID:s14119 siRNA (Thermo Fisher Scientific) was incubated with lipofectamine RNAiMAX (Thermo Fisher Scientific) and Opti-Mem I Reduced Serum Medium (Thermo Fisher Scientific) according to manufacturer's instructions.

After 72 hours of incubation with RNAi, RNA was extracted from WERI-Rb1 cells using the Zymo Direct-zol RNA Microprep Kit (Zymo Research) according to manufacturer's instructions. RNA concentration was determined on a Nanodrop One (Thermo Fisher Scientific) and equal concentrations of RNA for each sample were used to generate cDNA using the RETROscript Reverse Transcription Kit (Thermo Fisher Scientific) according to manufacturer's instructions.

Quantitative Real-Time PCR was performed using TaqMan Gene Expression MasterMix (Thermo Fisher Scientific) on a StepOnePlus Real-Time PCR System (Applied Biosystems). Relative gene expression levels were determined for the genes *THRβ* (Hs00230861_m1 TaqMan probe from Thermo Fisher Scientific), and *OPN1LW & OPN1MW* (could not discriminate) (Hs01912094_s1 TaqMan probe from Thermo Fisher Scientific) and normalized to *GAPDH* (Hs02758991_g1 TaqMan probe from Thermo Fisher Scientific) using the delta-delta ct approach. For each condition, three biological replicates were performed and three technical replicates were run on the same plate for each primer set. Fold change was calculated relative to an siRNA negative control sample lacking T3.

Measurements and Quantification

Measurements of retinal area and cell morphology were done using imageJ software. Quantifications and statistics (except for RNA-seq data) were done in GraphPad Prism, with a significance cutoff of 0.01. Statistical tests are listed in figure legends. All error bars represent the SEM.

RNA-Seq time course analysis

Expression levels were quantified using Kallisto (version 0.34.1) with the following parameters: "-b 100 -l 200 -s 10 -t 20 --single". The Gencode release 28 comprehensive annotation was used as the reference transcriptome (*60*). Transcripts per million (TPM) values (**Table S1**) were then used to generate graphs in Prism and heatmaps in R using ggplot2. The distributions of transcripts were plotted to identify the best low TPM cutoff (**Fig. S5A**). The threshold was determined to be 0.7 Log(TPM+1), i.e. 5 TPM, and this value was used as an

inflection point for heatmap. Heatmaps for **Fig. S3A-C** were made similarly, using CPM values from Hoshino, *et. al* (**Fig. S5B**) (*23*).

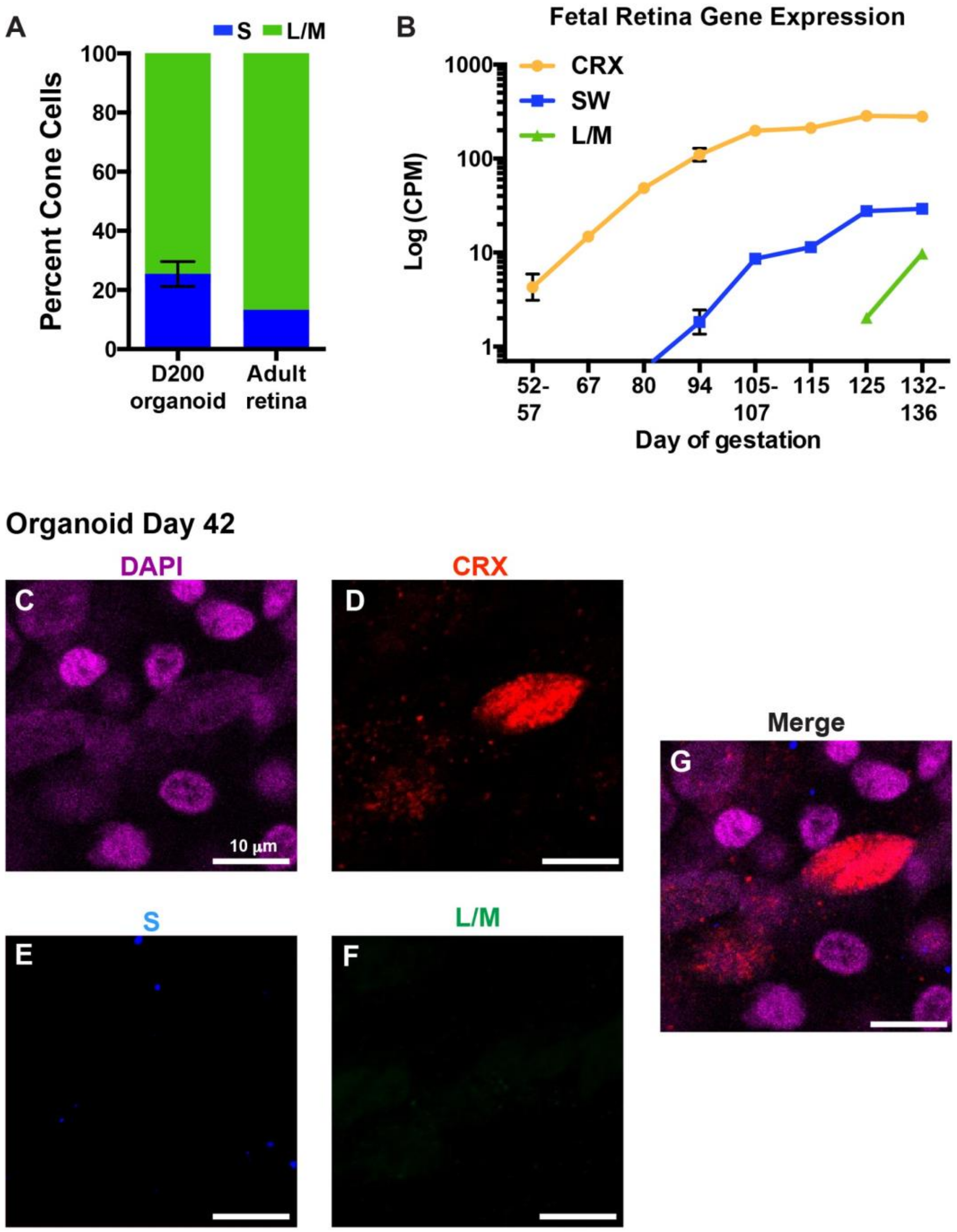

Supplemental Figure 1. *CRX* expression precedes *S-opsin* and *L/M-opsin* expression

A) Percent of S and L/M cones in day 200 organoid (n=7) and adult retina age 53 (n=1).

B) CPM values from Hoshino et. al (*23*) for *CRX*, *S*, and *L/M.*

C-G) Antibody staining for DAPI (magenta), CRX (red), S-opsin (blue), L/M-opsin (green).

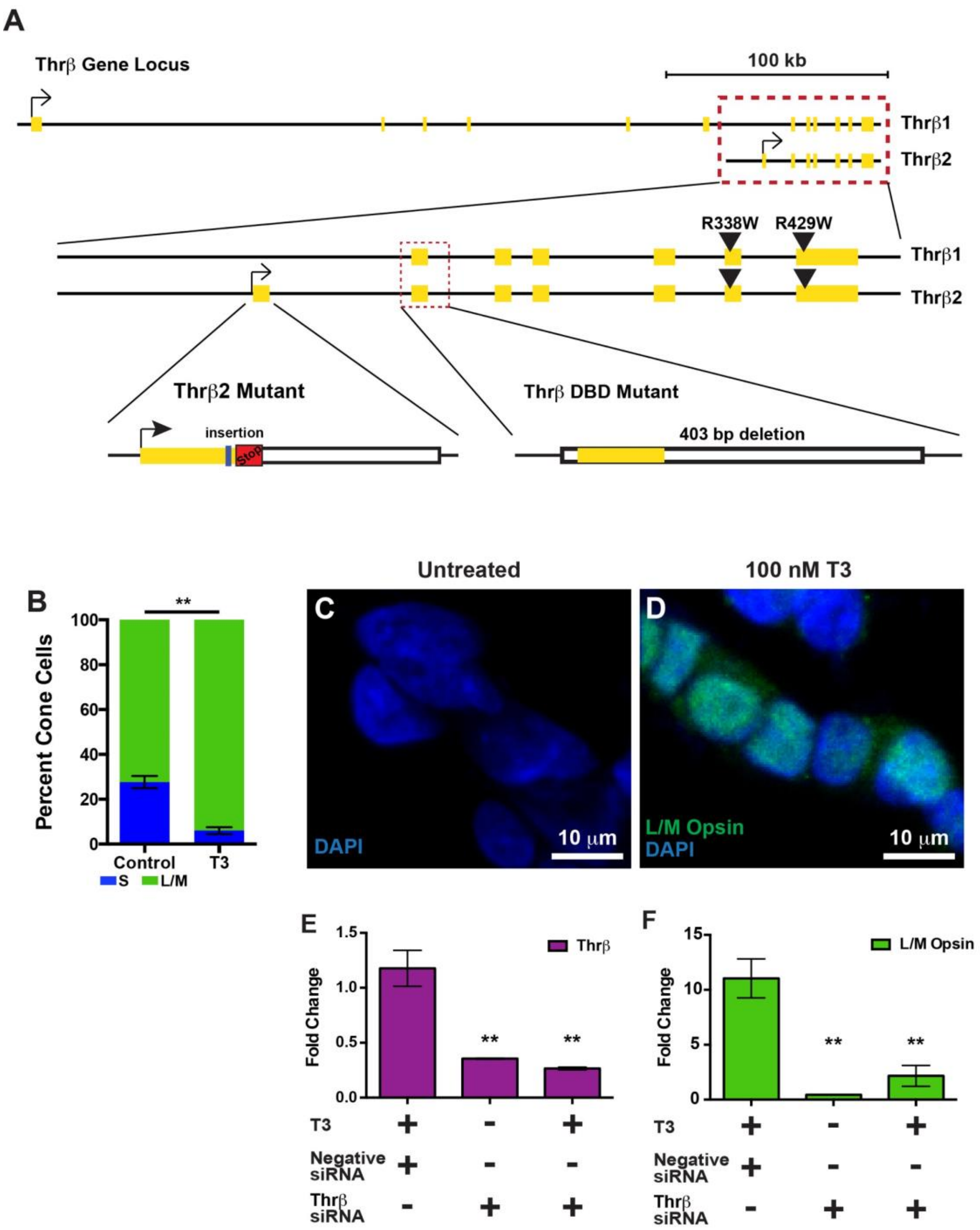

Supplemental Figure 2. T3 signals through *Thrβ* to suppress S fate and promote L/M fate

A) Gene diagram of *Thrβ1* and *Thrβ2* locus and homozygous deletions made with CRISPR/Cas9. Arrow heads represent the point mutations described in Weiss et. al (*30*). The individual was trans-heterozygous for these mutations.
B) Quantification of iPSC-derived organoids treated from day 20-200 of differentiation with 20nM T3. (Control, n=3; T3, n=3; student's t-test, P<0.001)
C-D) Fluorescence image of WERI-Rb1 cells stained with DAPI (blue) and antibody against L/M-opsin (green).

C) Untreated WERI-Rb1 cells.
D) WERI-Rb1 cells treated for 4 days with 100 nM T3.
E-F) siRNA knockdown of *Thrβ* in WERI-Rb1 cells, *Thrβ* and *L/M-opsin* analyzed by qPCR.
E) qPCR results of TaqMan probes for *Thrβ* (n=3 biological replicates. Tukey's multiple comparisons test: Negative siRNA + T3 vs Thrβ siRNA –T3, P<0.01; Negative siRNA+T3 vs Thrβ siRNA + T3, P<0.01).
F) qPCR results of TaqMan probes for *L/M-opsins* (n=3 biological replicates. Tukey's multiple comparisons test: Negative siRNA + T3 vs Thrβ siRNA –T3, P<0.01; Negative siRNA+T3 vs Thrβ siRNA + T3, P<0.01).

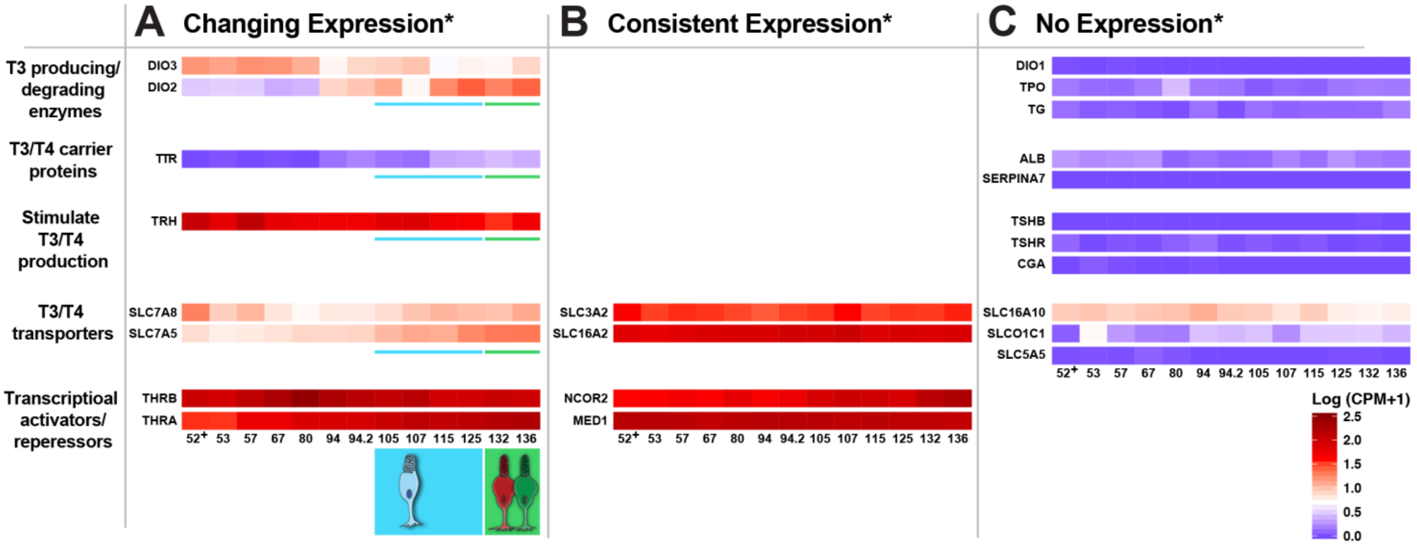

*Genes are categorized as in Figure 4A-C for consistency.
+ This data set is from fetal tissue between day 52 and 54 of gestation.

Supplemental Figure 3. Expression of thyroid hormone regulators in developing human retinas
A-C) Heat maps of Log(Counts per Kilobase Million (CPM) + 1) values for genes displayed in **Fig. 4A-C**. Numbers at the bottom of heat maps indicate fetal age in days. Genes are categorized as in **Fig. 4A-C** for consistency. The gene expression patterns in developing fetal tissue are similar to the patterns observed in developing organoids.

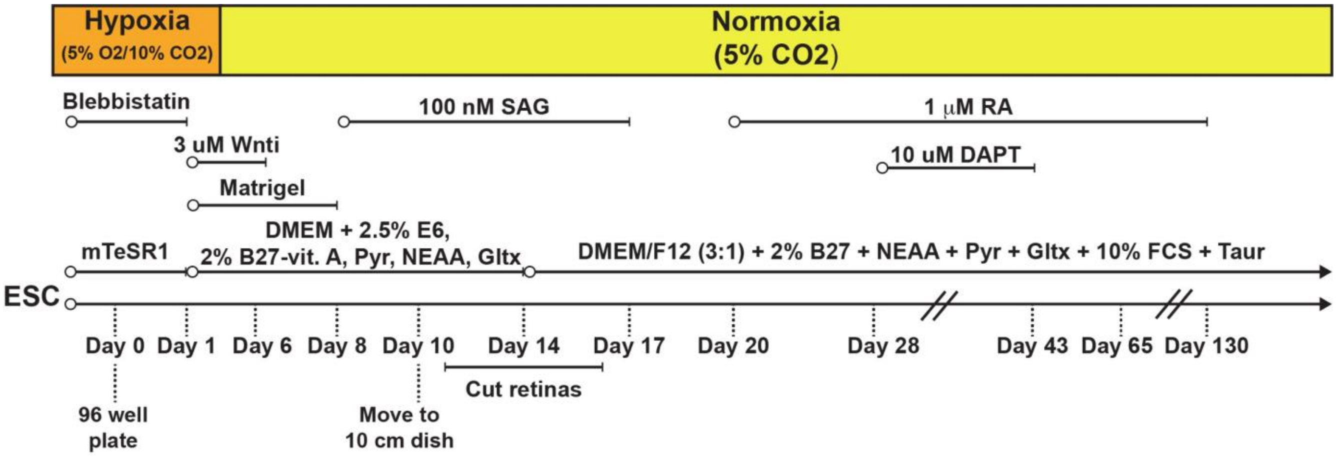

Supplemental Figure 4. Differentiation protocol for retinal organoids
Abbreviations are as follows:
Gltx = Glutamax
Pyr = Sodium Pyruvate
RA = Retinoic Acid
SAG = Smoothend Agonist
Taur = Taurine
Vit. A = vitamin A
Wnti = Wnt inhibitor

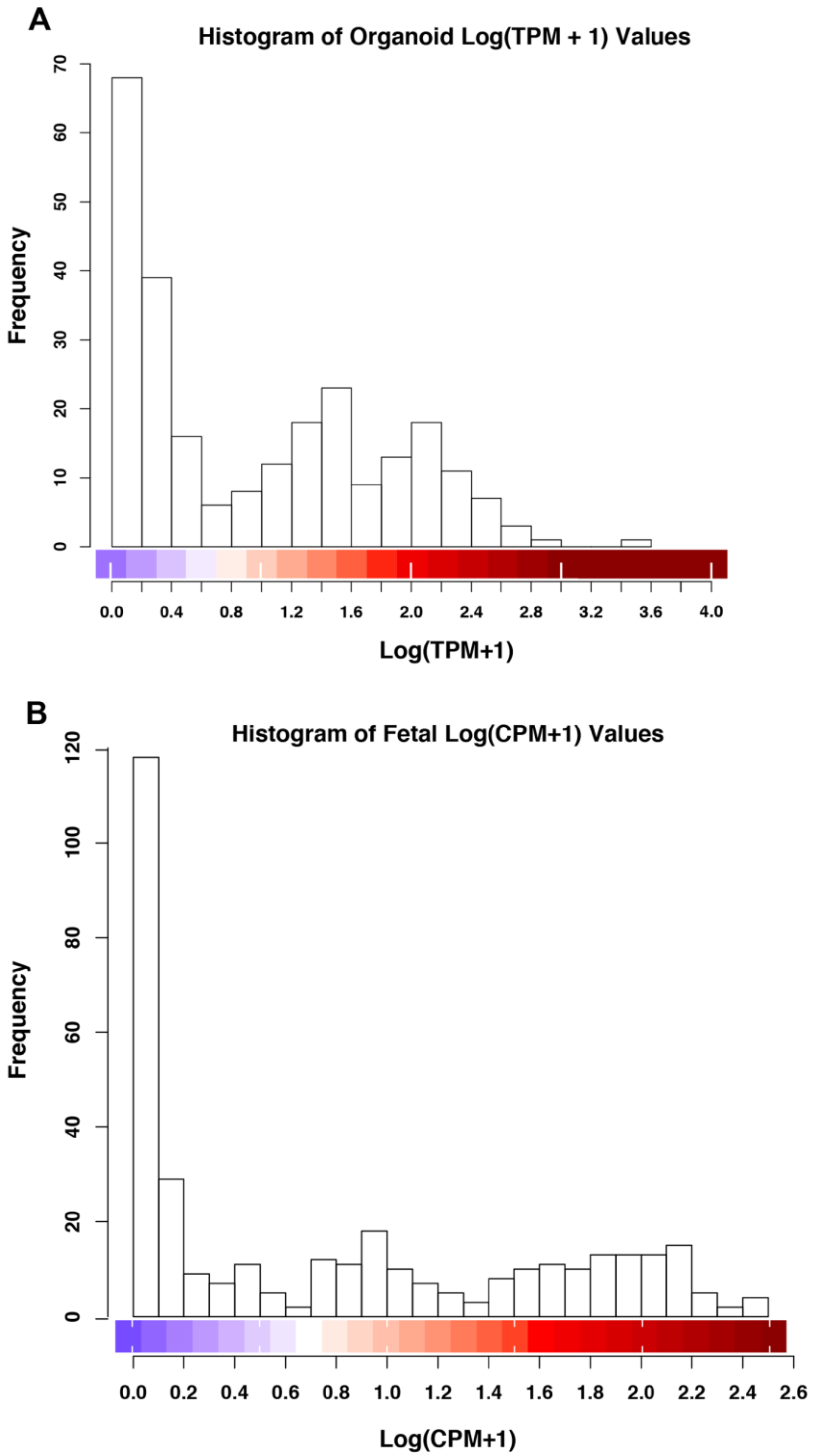

Supplemental Figure 5. Histogram of expression values used to identify the inflection point in the heat map of transcript expression
A) Histogram of TPM values used to identify the inflection point in the heat map included in **Fig. 4A-C**.
B) Histogram of CPM values from Hoshino et. al (*23*) to identify the inflection point in the heat map included in **Fig. S3A-C**.

References and Notes

1. K. Viets, K. Eldred, R. J. Johnston Jr., Mechanisms of photoreceptor patterning in vertebrates and invertebrates. *Trends Genet.* **32**, 638–659 (2016). doi:10.1016/j.tig.2016.07.004 Medline

2. J. Nathans, D. Thomas, D. S. Hogness, Molecular genetics of human color vision: The genes encoding blue, green, and red pigments. *Science* **232**, 193–202 (1986). doi:10.1126/science.2937147 Medline

3. D. Vollrath, J. Nathans, R. W. Davis, Tandem array of human visual pigment genes at Xq28. *Science* **240**, 1669–1672 (1988). doi:10.1126/science.2837827 Medline

4. Y. Wang, J. P. Macke, S. L. Merbs, D. J. Zack, B. Klaunberg, J. Bennett, J. Gearhart, J. Nathans, A locus control region adjacent to the human red and green visual pigment genes. *Neuron* **9**, 429–440 (1992). doi:10.1016/0896-6273(92)90181-C Medline

5. P. M. Smallwood, Y. Wang, J. Nathans, Role of a locus control region in the mutually exclusive expression of human red and green cone pigment genes. *Proc. Natl. Acad. Sci. U.S.A.* **99**, 1008–1011 (2002). doi:10.1073/pnas.022629799 Medline

6. Y. Wang, P. M. Smallwood, M. Cowan, D. Blesh, A. Lawler, J. Nathans, Mutually exclusive expression of human red and green visual pigment-reporter transgenes occurs at high frequency in murine cone photoreceptors. *Proc. Natl. Acad. Sci. U.S.A.* **96**, 5251–5256 (1999). doi:10.1073/pnas.96.9.5251 Medline

7. J. Nathans, C. M. Davenport, I. H. Maumenee, R. A. Lewis, J. F. Hejtmancik, M. Litt, E. Lovrien, R. Weleber, B. Bachynski, F. Zwas, et, Molecular genetics of human blue cone monochromacy. *Science* **245**, 831–838 (1989). doi:10.1126/science.2788922 Medline

8. A. S. Ladekjaer-Mikkelsen, T. Rosenberg, A. L. Jørgensen, A new mechanism in blue cone monochromatism. *Hum. Genet.* **98**, 403–408 (1996). doi:10.1007/s004390050229 Medline

9. E. J. Patterson, M. Wilk, C. S. Langlo, M. Kasilian, M. Ring, R. B. Hufnagel, A. M. Dubis, J. J. Tee, A. Kalitzeos, J. C. Gardner, Z. M. Ahmed, R. A. Sisk, M. Larsen, S. Sjoberg, T. B. Connor, A. Dubra, J. Neitz, A. J. Hardcastle, M. Neitz, M. Michaelides, J. Carroll, Cone photoreceptor structure in patients with X-linked cone dysfunction and red-green color vision deficiency. *Invest. Ophthalmol. Vis. Sci.* **57**, 3853–3863 (2016). doi:10.1167/iovs.16-19608 Medline

10. T. Nakano, S. Ando, N. Takata, M. Kawada, K. Muguruma, K. Sekiguchi, K. Saito, S. Yonemura, M. Eiraku, Y. Sasai, Self-formation of optic cups and storable stratified neural retina from human ESCs. *Cell Stem Cell* **10**, 771–785 (2012). doi:10.1016/j.stem.2012.05.009 Medline

11. X. Zhong, C. Gutierrez, T. Xue, C. Hampton, M. N. Vergara, L.-H. Cao, A. Peters, T. S. Park, E. T. Zambidis, J. S. Meyer, D. M. Gamm, K.-W. Yau, M. V. Canto-Soler, Generation of three-dimensional retinal tissue with functional photoreceptors from human iPSCs. *Nat. Commun.* **5**, 4047 (2014). doi:10.1038/ncomms5047 Medline

12. K. J. Wahlin, J. A. Maruotti, S. R. Sripathi, J. Ball, J. M. Angueyra, C. Kim, R. Grebe, W. Li, B. W. Jones, D. J. Zack, Photoreceptor outer segment-like structures in long-term 3D

retinas from human pluripotent stem cells. *Sci. Rep.* **7**, 766 (2017). doi:10.1038/s41598-017-00774-9 Medline

13. R. Kaewkhaw, K. D. Kaya, M. Brooks, K. Homma, J. Zou, V. Chaitankar, M. Rao, A. Swaroop, Transcriptome dynamics of developing photoreceptors in three-dimensional retina cultures recapitulates temporal sequence of human cone and rod differentiation revealing cell surface markers and gene networks. *Stem Cells* **33**, 3504–3518 (2015). doi:10.1002/stem.2122 Medline

14. M. J. Phillips, P. Jiang, S. Howden, P. Barney, J. Min, N. W. York, L.-F. Chu, E. E. Capowski, A. Cash, S. Jain, K. Barlow, T. Tabassum, R. Stewart, B. R. Pattnaik, J. A. Thomson, D. M. Gamm, A novel approach to single cell RNA-sequence analysis facilitates in silico gene reporting of human pluripotent stem cell-derived retinal cell types. *Stem Cells* **36**, 313–324 (2018). doi:10.1002/stem.2755 Medline

15. A. Artero Castro, D. Lukovic, P. Jendelova, S. Erceg, Concise review: Human induced pluripotent stem cell models of retinitis pigmentosa. *Stem Cells* **36**, 474–481 (2018). doi:10.1002/stem.2783 Medline

16. T. Furukawa, E. M. Morrow, C. L. Cepko, Crx, a novel otx-like homeobox gene, shows photoreceptor-specific expression and regulates photoreceptor differentiation. *Cell* **91**, 531–541 (1997). doi:10.1016/S0092-8674(00)80439-0 Medline

17. C. L. Freund, C. Y. Gregory-Evans, T. Furukawa, M. Papaioannou, J. Looser, L. Ploder, J. Bellingham, D. Ng, J.-A. S. Herbrick, A. Duncan, S. W. Scherer, L.-C. Tsui, A. Loutradis-Anagnostou, S. G. Jacobson, C. L. Cepko, S. S. Bhattacharya, R. R. McInnes, Cone-rod dystrophy due to mutations in a novel photoreceptor-specific homeobox gene (CRX) essential for maintenance of the photoreceptor. *Cell* **91**, 543–553 (1997). doi:10.1016/S0092-8674(00)80440-7 Medline

18. S. Chen, Q.-L. Wang, Z. Nie, H. Sun, G. Lennon, N. G. Copeland, D. J. Gilbert, N. A. Jenkins, D. J. Zack, Crx, a novel Otx-like paired-homeodomain protein, binds to and transactivates photoreceptor cell-specific genes. *Neuron* **19**, 1017–1030 (1997). doi:10.1016/S0896-6273(00)80394-3 Medline

19. C. A. Curcio, K. A. Allen, K. R. Sloan, C. L. Lerea, J. B. Hurley, I. B. Klock, A. H. Milam, Distribution and morphology of human cone photoreceptors stained with anti-blue opsin. *J. Comp. Neurol.* **312**, 610–624 (1991). doi:10.1002/cne.903120411 Medline

20. A. Hendrickson, D. Drucker, The development of parafoveal and mid-peripheral human retina. *Behav. Brain Res.* **49**, 21–31 (1992). doi:10.1016/S0166-4328(05)80191-3 Medline

21. M. Xiao, A. Hendrickson, Spatial and temporal expression of short, long/medium, or both opsins in human fetal cones. *J. Comp. Neurol.* **425**, 545–559 (2000). doi:10.1002/1096-9861(20001002)425:4<545:AID-CNE6>3.0.CO;2-3 Medline

22. C. A. Curcio, K. R. Sloan, R. E. Kalina, A. E. Hendrickson, Human photoreceptor topography. *J. Comp. Neurol.* **292**, 497–523 (1990). doi:10.1002/cne.902920402 Medline

23. A. Hoshino, R. Ratnapriya, M. J. Brooks, V. Chaitankar, M. S. Wilken, C. Zhang, M. R. Starostik, L. Gieser, A. La Torre, M. Nishio, O. Bates, A. Walton, O. Bermingham-McDonogh, I. A. Glass, R. O. L. Wong, A. Swaroop, T. A. Reh, Molecular anatomy of

the developing human retina. *Dev. Cell* **43**, 763–779.e4 (2017). doi:10.1016/j.devcel.2017.10.029 Medline

24. L. Ng, J. B. Hurley, B. Dierks, M. Srinivas, C. Saltó, B. Vennström, T. A. Reh, D. Forrest, A thyroid hormone receptor that is required for the development of green cone photoreceptors. *Nat. Genet.* **27**, 94–98 (2001). doi:10.1038/83829 Medline

25. M. R. Roberts, M. Srinivas, D. Forrest, G. Morreale de Escobar, T. A. Reh, Making the gradient: Thyroid hormone regulates cone opsin expression in the developing mouse retina. *Proc. Natl. Acad. Sci. U.S.A.* **103**, 6218–6223 (2006). doi:10.1073/pnas.0509981103 Medline

26. M. L. Applebury, F. Farhangfar, M. Glösmann, K. Hashimoto, K. Kage, J. T. Robbins, N. Shibusawa, F. E. Wondisford, H. Zhang, Transient expression of thyroid hormone nuclear receptor TRbeta2 sets S opsin patterning during cone photoreceptor genesis. *Dev. Dyn.* **236**, 1203–1212 (2007). doi:10.1002/dvdy.21155 Medline

27. S. C. Suzuki, A. Bleckert, P. R. Williams, M. Takechi, S. Kawamura, R. O. L. Wong, Cone photoreceptor types in zebrafish are generated by symmetric terminal divisions of dedicated precursors. *Proc. Natl. Acad. Sci. U.S.A.* **110**, 15109–15114 (2013). doi:10.1073/pnas.1303551110 Medline

28. M. Sjöberg, B. Vennström, D. Forrest, Thyroid hormone receptors in chick retinal development: Differential expression of mRNAs for alpha and N-terminal variant beta receptors. *Development* **114**, 39–47 (1992). Medline

29. J. M. Trimarchi, S. Harpavat, N. A. Billings, C. L. Cepko, Thyroid hormone components are expressed in three sequential waves during development of the chick retina. *BMC Dev. Biol.* **8**, 101 (2008). doi:10.1186/1471-213X-8-101 Medline

30. A. H. Weiss, J. P. Kelly, D. Bisset, S. S. Deeb, Reduced L- and M- and increased S-cone functions in an infant with thyroid hormone resistance due to mutations in the THRβ2 gene. *Ophthalmic Genet.* **33**, 187–195 (2012). doi:10.3109/13816810.2012.681096 Medline

31. H. H. Samuels, J. S. Tsai, J. Casanova, F. Stanley, Thyroid hormone action: In vitro characterization of solubilized nuclear receptors from rat liver and cultured GH1 cells. *J. Clin. Invest.* **54**, 853–865 (1974). doi:10.1172/JCI107825 Medline

32. A. Glaschke, M. Glösmann, L. Peichl, Developmental changes of cone opsin expression but not retinal morphology in the hypothyroid Pax8 knockout mouse. *Invest. Ophthalmol. Vis. Sci.* **51**, 1719–1727 (2010). doi:10.1167/iovs.09-3592 Medline

33. A. Glaschke, J. Weiland, D. Del Turco, M. Steiner, L. Peichl, M. Glösmann, Thyroid hormone controls cone opsin expression in the retina of adult rodents. *J. Neurosci.* **31**, 4844–4851 (2011). doi:10.1523/JNEUROSCI.6181-10.2011 Medline

34. Y. Liu, L. Fu, D. G. Chen, S. S. Deeb, Identification of novel retinal target genes of thyroid hormone in the human WERI cells by expression microarray analysis. *Vision Res.* **47**, 2314–2326 (2007). doi:10.1016/j.visres.2007.04.023 Medline

35. A. Schroeder, R. Jimenez, B. Young, M. L. Privalsky, The ability of thyroid hormone receptors to sense t4 as an agonist depends on receptor isoform and on cellular cofactors. *Mol. Endocrinol.* **28**, 745–757 (2014). doi:10.1210/me.2013-1335 Medline

36. M. Dentice, A. Marsili, A. Zavacki, P. R. Larsen, D. Salvatore, The deiodinases and the control of intracellular thyroid hormone signaling during cellular differentiation. *Biochim. Biophys. Acta* **1830**, 3937–3945 (2013). doi:10.1016/j.bbagen.2012.05.007 Medline

37. V. M. Darras, A. M. Houbrechts, S. L. Van Herck, Intracellular thyroid hormone metabolism as a local regulator of nuclear thyroid hormone receptor-mediated impact on vertebrate development. *Biochim. Biophys. Acta* **1849**, 130–141 (2015). doi:10.1016/j.bbagrm.2014.05.004 Medline

38. L. Ng, A. Lyubarsky, S. S. Nikonov, M. Ma, M. Srinivas, B. Kefas, D. L. St Germain, A. Hernandez, E. N. Pugh Jr., D. Forrest, Type 3 deiodinase, a thyroid-hormone-inactivating enzyme, controls survival and maturation of cone photoreceptors. *J. Neurosci.* **30**, 3347–3357 (2010). doi:10.1523/JNEUROSCI.5267-09.2010 Medline

39. P. J. Bonezzi, M. E. Stabio, J. M. Renna, The development of mid-wavelength photoresponsivity in the mouse retina. *Curr. Eye Res.* **43**, 666–673 (2018). doi:10.1080/02713683.2018.1433859 Medline

40. E. Bagci, M. Heijlen, L. Vergauwen, A. Hagenaars, A. M. Houbrechts, C. V. Esguerra, R. Blust, V. M. Darras, D. Knapen, Deiodinase knockdown during early zebrafish development affects growth, development, energy metabolism, motility and phototransduction. *PLOS ONE* **10**, e0123285 (2015). doi:10.1371/journal.pone.0123285 Medline

41. C. Guo, X. Chen, H. Song, M. A. Maynard, Y. Zhou, A. V. Lobanov, V. N. Gladyshev, J. J. Ganis, D. Wiley, R. H. Jugo, N. Y. Lee, L. A. Castroneves, L. I. Zon, T. S. Scanlan, H. A. Feldman, S. A. Huang, Intrinsic expression of a multiexon type 3 deiodinase gene controls zebrafish embryo size. *Endocrinology* **155**, 4069–4080 (2014). doi:10.1210/en.2013-2029 Medline

42. S. L. Bruhn, C. L. Cepko, Development of the pattern of photoreceptors in the chick retina. *J. Neurosci.* **16**, 1430–1439 (1996). doi:10.1523/JNEUROSCI.16-04-01430.1996 Medline

43. A. C. Bianco, D. Salvatore, B. Gereben, M. J. Berry, P. R. Larsen, Biochemistry, cellular and molecular biology, and physiological roles of the iodothyronine selenodeiodinases. *Endocr. Rev.* **23**, 38–89 (2002). doi:10.1210/edrv.23.1.0455 Medline

44. D. S. Sharlin, T. J. Visser, D. Forrest, Developmental and cell-specific expression of thyroid hormone transporters in the mouse cochlea. *Endocrinology* **152**, 5053–5064 (2011). doi:10.1210/en.2011-1372 Medline

45. T. C. Lee, D. Almeida, N. Claros, D. H. Abramson, D. Cobrinik, Cell cycle-specific and cell type-specific expression of Rb in the developing human retina. *Invest. Ophthalmol. Vis. Sci.* **47**, 5590–5598 (2006). doi:10.1167/iovs.06-0063 Medline

46. E. J. Barrett, in *Medical Physiology, 2e Updated Edition,* W. B. E. Boulpaep, Ed. (Elsevier, Inc., Philadelphia, PA, 2012), chap. 49.

47. S. R. Dubovy, M. P. Fernandez, J. J. Echegaray, N. L. Block, N. Unoki, R. Perez, I. Vidaurre, R. K. Lee, M. Nadji, A. V. Schally, Expression of hypothalamic neurohormones and their receptors in the human eye. *Oncotarget* **8**, 66796–66814 (2017). doi:10.18632/oncotarget.18358 Medline

48. E. Martino, M. Nardi, G. Vaudagna, S. Simonetti, A. Cilotti, A. Pinchera, G. Venturi, H. Seo, L. Baschieri, Thyrotropin-releasing hormone-like material in human retina. *J. Endocrinol. Invest.* **3**, 267–271 (1980). doi:10.1007/BF03348274 Medline

49. J. Rovet, N. Simic, The role of transient hypothyroxinemia of prematurity in development of visual abilities. *Semin. Perinatol.* **32**, 431–437 (2008). doi:10.1053/j.semperi.2008.09.009 Medline

50. N. Simic, C. Westall, E. V. Astzalos, J. Rovet, Visual abilities at 6 months in preterm infants: Impact of thyroid hormone deficiency and neonatal medical morbidity. *Thyroid* **20**, 309–315 (2010). doi:10.1089/thy.2009.0128 Medline

51. S. A. Yassin, A. J. Al-Dawood, W. M. Al-Zamil, M. A. Al-Ghamdi, Z. N. Al-Khudairy, Comparative study of visual dysfunctions in 6-10-year-old very preterm- and full-term-born children. *Int. Ophthalmol.* (2018). doi:10.1007/s10792-018-0959-2 Medline

52. H. J. Dowdeswell, A. M. Slater, J. Broomhall, J. Tripp, Visual deficits in children born at less than 32 weeks' gestation with and without major ocular pathology and cerebral damage. *Br. J. Ophthalmol.* **79**, 447–452 (1995). doi:10.1136/bjo.79.5.447 Medline

53. R. A. Pearson, A. C. Barber, M. Rizzi, C. Hippert, T. Xue, E. L. West, Y. Duran, A. J. Smith, J. Z. Chuang, S. A. Azam, U. F. O. Luhmann, A. Benucci, C. H. Sung, J. W. Bainbridge, M. Carandini, K.-W. Yau, J. C. Sowden, R. R. Ali, Restoration of vision after transplantation of photoreceptors. *Nature* **485**, 99–103 (2012). doi:10.1038/nature10997 Medline

54. A. O. Barnea-Cramer, W. Wang, S.-J. Lu, M. S. Singh, C. Luo, H. Huo, M. E. McClements, A. R. Barnard, R. E. MacLaren, R. Lanza, Function of human pluripotent stem cell-derived photoreceptor progenitors in blind mice. *Sci. Rep.* **6**, 29784 (2016). doi:10.1038/srep29784 Medline

55. D. A. Lamba, J. Gust, T. A. Reh, Transplantation of human embryonic stem cell-derived photoreceptors restores some visual function in Crx-deficient mice. *Cell Stem Cell* **4**, 73–79 (2009). doi:10.1016/j.stem.2008.10.015 Medline

56. B. A. Tucker, I.-H. Park, S. D. Qi, H. J. Klassen, C. Jiang, J. Yao, S. Redenti, G. Q. Daley, M. J. Young, Transplantation of adult mouse iPS cell-derived photoreceptor precursors restores retinal structure and function in degenerative mice. *PLOS ONE* **6**, e18992 (2011). doi:10.1371/journal.pone.0018992 Medline

57. J. Harrow *et al.*, GENCODE: The reference human genome annotation for The ENCODE Project. *Genome Res.* **22**, 1760–1774 (2012).

58. N. S. Bhise, K. J. Wahlin, D. J. Zack, J. J. Green, Evaluating the potential of poly(beta-amino ester) nanoparticles for reprogramming human fibroblasts to become induced pluripotent stem cells. *Int. J. Nanomedicine* **8**, 4641–4658 (2013). Medline

59. V. M. Sluch, X. Chamling, M. M. Liu, C. A. Berlinicke, J. Cheng, K. L. Mitchell, D. S. Welsbie, D. J. Zack, Enhanced stem cell differentiation and immunopurification of genome engineered human retinal ganglion cells. *Stem Cells Transl. Med.* **6**, 1972–1986 (2017). doi:10.1002/sctm.17-0059 Medline

60. J. Harrow, A. Frankish, J. M. Gonzalez, E. Tapanari, M. Diekhans, F. Kokocinski, B. L. Aken, D. Barrell, A. Zadissa, S. Searle, I. Barnes, A. Bignell, V. Boychenko, T. Hunt, M. Kay, G. Mukherjee, J. Rajan, G. Despacio-Reyes, G. Saunders, C. Steward, R. Harte, M. Lin, C. Howald, A. Tanzer, T. Derrien, J. Chrast, N. Walters, S. Balasubramanian, B. Pei, M. Tress, J. M. Rodriguez, I. Ezkurdia, J. van Baren, M. Brent, D. Haussler, M. Kellis, A. Valencia, A. Reymond, M. Gerstein, R. Guigó, T. J. Hubbard, GENCODE: The reference human genome annotation for The ENCODE Project. *Genome Res.* **22**, 1760–1774 (2012). doi:10.1101/gr.135350.111 Medline
